# (3*S*,4*S*,5*S*)-4-Hydr­oxy-3-methyl-5-[(2*S*,3*R*)-3-methyl­pent-4-en-2-yl]-4,5-dihydro­furan-2(3*H*)-one

**DOI:** 10.1107/S1600536808026998

**Published:** 2008-08-30

**Authors:** Annika Gille, Markus Schürmann, Hans Preut, Martin Hiersemann

**Affiliations:** a Fakultät Chemie, Technische Universität Dortmund, Otto-Hahn-Str. 6, 44221 Dortmund, Germany

## Abstract

The title compound, C_11_H_18_O_3_, was synthesized to prove the relative configuration of the corresponding acyclic C1—C8 stereopentade. Mol­ecules are linked *via* O—H⋯O hydrogen bonds, forming a chain along the *b* axis.

## Related literature

For related literature, see: Abraham *et al.* (2004*a* ▶,*b* ▶); Corey & Snider (1972 ▶); Evans *et al.* (1981 ▶, 1999 ▶); Körner & Hiersemann (2006 ▶, 2007 ▶); Pollex & Hiersemann (2005 ▶).
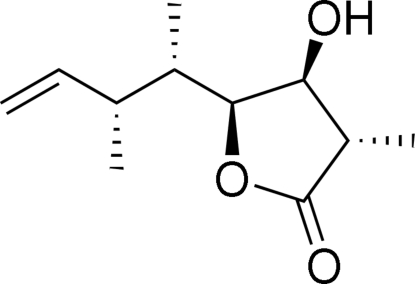


## Experimental

### 

#### Crystal data

C_11_H_18_O_3_
*M* *_r_* = 198.25
Orthorhombic, 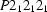

*a* = 5.4414 (14) Å
*b* = 10.132 (2) Å
*c* = 20.975 (8) Å
*V* = 1156.4 (6) Å^3^
*Z* = 4
Mo *K*α radiation
μ = 0.08 mm^−1^
*T* = 291 (1) K
0.36 × 0.06 × 0.02 mm

#### Data collection

Nonius KappaCCD diffractometer
Absorption correction: none
7554 measured reflections
1223 independent reflections
346 reflections with *I* > 2σ(*I*)
*R*_int_ = 0.048

#### Refinement

*R*[*F* ^2^ > 2σ(*F* ^2^)] = 0.036
*wR*(*F* ^2^) = 0.087
*S* = 0.97
1223 reflections
131 parameters
H-atom parameters constrained
Δρ_max_ = 0.09 e Å^−3^
Δρ_min_ = −0.13 e Å^−3^

### 

Data collection: *COLLECT* (Nonius, 1998 ▶); cell refinement: *DENZO* and *SCALEPACK* (Otwinowski & Minor, 1997 ▶); data reduction: *DENZO* and *SCALEPACK*; program(s) used to solve structure: *SHELXS97* (Sheldrick, 2008 ▶); program(s) used to refine structure: *SHELXL97* (Sheldrick, 2008 ▶); molecular graphics: *SHELXTL-Plus* (Sheldrick, 2008 ▶); software used to prepare material for publication: *SHELXL97* and *PLATON* (Spek, 2003 ▶).

## Supplementary Material

Crystal structure: contains datablocks I, global. DOI: 10.1107/S1600536808026998/bt2768sup1.cif

Structure factors: contains datablocks I. DOI: 10.1107/S1600536808026998/bt2768Isup2.hkl

Additional supplementary materials:  crystallographic information; 3D view; checkCIF report

## Figures and Tables

**Table 1:** Hydrogen-bond geometry (Å, °)

*D*—H⋯*A*	*D*—H	H⋯*A*	*D*⋯*A*	*D*—H⋯*A*
O3—H3⋯O2^i^	0.82	2.02	2.798 (6)	158

Symmetry code: (i) 
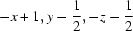
.

**Fig. 1.**
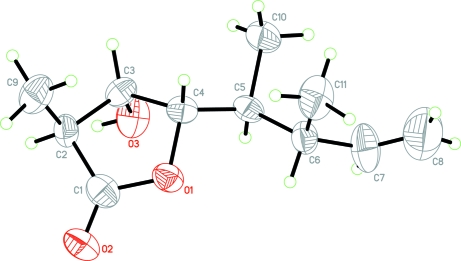
: The molecular structure of the title compound, showing the labelling of all non-H atoms. Displacement ellipsoids are shown at the 30% probability level.

## supplementary crystallographic information

### Comment

The title compound, (I), was synthesized using a catalytic asymmetric Claisen rearrangement (Abraham *et al.*, 2004*a*; Abraham *et al.*, 2004*b*; Pollex & Hiersemann, 2005; Körner & Hiersemann, 2006; Körner & Hiersemann, 2007), a diastereoselective reduction with K-Selectride (Körner & Hiersemann, 2006; Körner & Hiersemann, 2007), and an Evans aldol addition (Evans *et al.*, 1981). In order to verify the relative configuration of the obtained aldol adduct, 4-(*tert*-butyldimethylsilyloxy)-3-hydroxy-2,5,6-trimethyloct-7-enoyl)-4-isopropyloxazolidin-2-one, (II), a γ-lactone, (I), was prepared by removal of the silyl protecting group (Corey & Snider, 1972) and subsequent *in situ *lactonization. Fig. 1 depicts the structure of the isolated major diastereomer (I). The configuration of the chiral C atoms in (I) can be attributed to the stereochemical course of the Evans aldol addition (C3 *S* and C4 *S*), the diastereoselective reduction with K-Selectride (C5 *S*), and the catalytic asymmetric Claisen rearrangement (C(2) *S* and C(3) *R*) using the chiral Lewis acid [Cu{(*S*,*S*)-*tert*-Butyl-box}](H_2_O)_2_(SbF_6_)_2_ (Evans *et al.*, 1999).

### Experimental

The title compound, (I), was synthesized from the corresponding *syn*-aldol adduct, (II), using tetrabutylammonium fluoride (Corey & Snider, 1972) for the removal of the silyl protecting group. The subsequent lactonization proceeded *in situ*.

To a solution of diastereomerically pure (II) (50 mg, 0.113 mmol, 1.0 eq) in dry tetrahydrofuran (1 ml) was added TBAF (1 *M* in tetrahydrofuran, 0.34 ml, 3.0 eq) at 273 K. The mixture was stirred at 273 K for 25 min. The reaction was then quenched by the addition of sat. aqueous NaHCO_3_ solution. The phases were separated, and the aqueous phase was extracted with CH_2_Cl_2_. The combined organic layers were dried over MgSO_4_ and concentrated under reduced pressure. Flash chromatography (isohexane/ethyl acetate 20/1 to 10/1) afforded (I) as a single diastereomer and additionally a mixture of (I) and the minor diastereomer with an overall yield of 96% (21.4 mg, 0.108 mmol) as colourless crystals. Single crystals of (I) were obtained by vapor diffusion recrystallization technique from isohexane and ethyl acetate to yield colourless needles: mp 374 K; *R_f_* 0.28 (cyclohexane/ethyl acetate 2/1); ^1^H NMR (CDCl_3_, 400 MHz, δ): 0.83 (d, *J* = 7.0 Hz, 3H), 0.98 (d, *J* = 6.8 Hz, 3H), 1.30 (d, *J* = 7.8 Hz, 3H), 2.21 (dqd, *J* = 10.7, 7.0, 3.3 Hz, 1H), 2.64 (q, *J* = 7.8 Hz, 1H) overlapped by 2.61 - 2.76 (m, 1H), 4.14 (d, *J* = 3.4 Hz, 1H), 4.23 (dd, *J* = 10.7, 3.4 Hz, 1H), 5.04 (dd, ^3^*J*(*Z*) = 11.0 Hz, ^2^*J* = 1.5 Hz, 1H), 5.05 (dd, ^3^*J*(*E*) = 17.0 Hz, ^2^*J* = 1.5 Hz, 1H), 5.84 (ddd, ^3^*J*(*E*) = 17.0 Hz, ^3^*J*(*Z*) = 11.0 Hz, ^3^*J* = 6.3 Hz, 1H); ^13^C NMR (CDCl_3_, 100 MHz, δ): 9.8 (CH_3_), 12.5 (CH_3_), 13.7 (CH_3_), 35.6 (CH), 37.2 (CH), 46.5 (CH), 75.0 (CH), 83.9 (CH), 114.4 (CH_2_), 142.7 (CH), 179.1 (C); IR (cm^-1^): 3520(br,s) (ν O—H, OH in H-bridges), 3085(w) (ν C—H, olefin), 2975(m) 2940(m) 2885(s) 2855(w) (ν_as,s_ C—H, CH_2_, CH_3_, CH), 1755(*s*) (ν C=O, lactone), 1640(w) (ν C=C), 1455(m) (δ_as_ C—H, CH_3_, CH_2_), 1385(m) (δ_s_ C—H, CH_3_); Anal. Calcd. for C_11_H_18_O_3_: C, 66.6; H, 9.2; Found: C, 66.5; H, 9.3; [α]_D_^20^ -14.5 (c 0.775, CHCl_3_).

### Refinement

The H atoms were geometrically placed (C-H = 0.93-0.98, O-H = 0.82 Å) and refined as riding with U_iso_(H) = 1.2_eq_(C, O) or 1.5U_eq_(methyl C).

### Crystal data

**Table:**

C_11_H_18_O_3_	*F*_000_ = 432
*M**_r_* = 198.25	*D*_x_ = 1.139 Mg m^−^^3^
Orthorhombic, *P*2_1_2_1_2_1_	Mo *K*α radiation λ = 0.71073 Å
Hall symbol: P 2ac 2ab	Cell parameters from 7554 reflections
*a* = 5.4414 (14) Å	θ = 2.8–25.0º
*b* = 10.132 (2) Å	µ = 0.08 mm^−^^1^
*c* = 20.975 (8) Å	*T* = 291 (1) K
*V* = 1156.4 (6) Å^3^	Needle, colourless
*Z* = 4	0.36 × 0.06 × 0.02 mm

### Data collection

**Table:**

Nonius KappaCCD diffractometer	1223 independent reflections
Radiation source: fine-focus sealed tube	346 reflections with *I* > 2σ(*I*)
Monochromator: graphite	*R*_int_ = 0.048
Detector resolution: 19 vertical, 18 horizontal pixels mm^-1^	θ_max_ = 25.0º
*T* = 291(1) K	θ_min_ = 2.8º
111 frames via ω–rotation (Δω=2%) and two times 180 s per frame (three sets at different κ–angles) scans	*h* = −6→6
Absorption correction: none	*k* = −12→12
7554 measured reflections	*l* = −24→24

### Refinement

**Table:**

Refinement on *F*^2^	Secondary atom site location: difference Fourier map
Least-squares matrix: full	Hydrogen site location: inferred from neighbouring sites
*R*[*F*^2^ > 2σ(*F*^2^)] = 0.036	H-atom parameters constrained
*wR*(*F*^2^) = 0.087	[1.0 exp(5.65(sinθ/λ)^2^)]/[σ^2^(*F*_o_^2^)]
*S* = 0.97	(Δ/σ)_max_ = 0.007
1223 reflections	Δρ_max_ = 0.09 e Å^−^^3^
131 parameters	Δρ_min_ = −0.13 e Å^−^^3^
Primary atom site location: structure-invariant direct methods	Extinction correction: none

### Special details

**Table:**

Geometry. All e.s.d.'s (except the e.s.d. in the dihedral angle between two l.s. planes) are estimated using the full covariance matrix. The cell e.s.d.'s are taken into account individually in the estimation of e.s.d.'s in distances, angles and torsion angles; correlations between e.s.d.'s in cell parameters are only used when they are defined by crystal symmetry. An approximate (isotropic) treatment of cell e.s.d.'s is used for estimating e.s.d.'s involving l.s. planes.
Refinement. Refinement of *F*^2^ against ALL reflections. The weighted *R*-factor *wR* and goodness of fit *S* are based on *F*^2^, conventional *R*-factors *R* are based on *F*, with *F* set to zero for negative *F*^2^. The threshold expression of *F*^2^ > σ(*F*^2^) is used only for calculating *R*-factors(gt) *etc*. and is not relevant to the choice of reflections for refinement. *R*-factors based on *F*^2^ are statistically about twice as large as those based on *F*, and *R*- factors based on ALL data will be even larger.

### Fractional atomic coordinates and isotropic or equivalent isotropic displacement parameters (Å^2^)

**Table:**

	*x*	*y*	*z*	*U*_iso_*/*U*_eq_	
O1	0.1778 (7)	0.0092 (4)	−0.3153 (2)	0.0647 (11)	
O2	0.4408 (8)	0.0462 (4)	−0.2370 (2)	0.0971 (19)	
O3	0.3095 (10)	−0.2710 (5)	−0.3391 (2)	0.0890 (15)	
H3	0.4090	−0.3080	−0.3158	0.133*	
C1	0.2948 (12)	−0.0261 (7)	−0.2615 (3)	0.075 (2)	
C2	0.2076 (11)	−0.1595 (6)	−0.2396 (3)	0.0641 (19)	
H2A	0.3449	−0.2114	−0.2227	0.077*	
C3	0.1142 (11)	−0.2191 (6)	−0.3017 (3)	0.0635 (19)	
H3B	−0.0147	−0.2849	−0.2942	0.076*	
C4	0.0156 (11)	−0.0982 (5)	−0.3372 (3)	0.0596 (18)	
H4A	−0.1522	−0.0803	−0.3227	0.072*	
C5	0.0176 (10)	−0.1020 (6)	−0.4090 (3)	0.0578 (17)	
H5A	0.1854	−0.1235	−0.4225	0.069*	
C6	−0.0502 (11)	0.0338 (6)	−0.4390 (3)	0.069 (2)	
H6A	0.0627	0.0986	−0.4205	0.082*	
C7	0.0064 (15)	0.0286 (7)	−0.5094 (4)	0.107 (3)	
H7A	0.1591	−0.0070	−0.5193	0.129*	
C8	−0.1131 (18)	0.0629 (9)	−0.5549 (4)	0.170 (4)	
H8A	−0.2681	0.0996	−0.5491	0.205*	
H8B	−0.0501	0.0527	−0.5958	0.205*	
C9	0.0066 (12)	−0.1412 (5)	−0.1883 (3)	0.093 (2)	
H9A	−0.0491	−0.2261	−0.1740	0.140*	
H9B	−0.1291	−0.0932	−0.2062	0.140*	
H9C	0.0731	−0.0929	−0.1529	0.140*	
C10	−0.1534 (11)	−0.2165 (5)	−0.4322 (3)	0.085 (2)	
H10A	−0.0821	−0.2998	−0.4207	0.128*	
H10B	−0.1712	−0.2119	−0.4777	0.128*	
H10C	−0.3118	−0.2079	−0.4125	0.128*	
C11	−0.3096 (12)	0.0798 (6)	−0.4222 (3)	0.102 (3)	
H11A	−0.3376	0.1657	−0.4401	0.152*	
H11B	−0.3269	0.0840	−0.3767	0.152*	
H11C	−0.4273	0.0187	−0.4393	0.152*	

### Atomic displacement parameters (Å^2^)

**Table:**

	*U*^11^	*U*^22^	*U*^33^	*U*^12^	*U*^13^	*U*^23^
O1	0.064 (3)	0.056 (3)	0.074 (3)	−0.002 (3)	−0.011 (3)	−0.006 (2)
O2	0.091 (4)	0.110 (4)	0.091 (4)	−0.027 (3)	−0.026 (3)	−0.012 (3)
O3	0.105 (4)	0.080 (3)	0.083 (3)	0.034 (3)	0.005 (3)	0.002 (3)
C1	0.062 (6)	0.083 (6)	0.081 (6)	0.010 (5)	−0.004 (5)	−0.010 (5)
C2	0.073 (5)	0.065 (5)	0.054 (5)	0.008 (4)	−0.013 (4)	0.009 (3)
C3	0.072 (5)	0.048 (4)	0.071 (5)	0.000 (4)	0.009 (4)	0.005 (4)
C4	0.053 (5)	0.056 (4)	0.070 (5)	0.000 (4)	0.002 (4)	−0.006 (4)
C5	0.039 (4)	0.068 (4)	0.066 (5)	0.001 (4)	−0.002 (4)	−0.008 (4)
C6	0.072 (6)	0.073 (4)	0.061 (5)	0.005 (4)	−0.013 (4)	0.009 (4)
C7	0.133 (7)	0.106 (6)	0.082 (7)	0.050 (6)	−0.028 (6)	0.007 (5)
C8	0.233 (13)	0.159 (9)	0.119 (10)	−0.006 (8)	−0.019 (9)	0.019 (7)
C9	0.119 (7)	0.089 (5)	0.071 (6)	0.002 (5)	0.021 (6)	0.004 (4)
C10	0.094 (6)	0.078 (5)	0.084 (5)	−0.016 (4)	−0.015 (5)	−0.013 (4)
C11	0.085 (6)	0.111 (6)	0.109 (6)	0.048 (5)	0.007 (5)	0.021 (4)

### Geometric parameters (Å, °)

**Table:**

O1—C1	1.344 (6)	C6—C7	1.508 (8)
O1—C4	1.475 (6)	C6—C11	1.527 (6)
O2—C1	1.196 (6)	C6—H6A	0.9800
O3—C3	1.422 (6)	C7—C8	1.207 (8)
O3—H3	0.8200	C7—H7A	0.9300
C1—C2	1.504 (7)	C8—H8A	0.9300
C2—C3	1.523 (7)	C8—H8B	0.9300
C2—C9	1.546 (7)	C9—H9A	0.9600
C2—H2A	0.9800	C9—H9B	0.9600
C3—C4	1.531 (6)	C9—H9C	0.9600
C3—H3B	0.9800	C10—H10A	0.9600
C4—C5	1.506 (6)	C10—H10B	0.9600
C4—H4A	0.9800	C10—H10C	0.9600
C5—C6	1.557 (6)	C11—H11A	0.9600
C5—C10	1.564 (7)	C11—H11B	0.9600
C5—H5A	0.9800	C11—H11C	0.9600
			
C1—O1—C4	110.5 (5)	C7—C6—C5	108.4 (5)
C3—O3—H3	109.5	C11—C6—C5	113.3 (5)
O2—C1—O1	120.8 (7)	C7—C6—H6A	106.5
O2—C1—C2	129.0 (7)	C11—C6—H6A	106.5
O1—C1—C2	110.2 (6)	C5—C6—H6A	106.5
C1—C2—C3	101.6 (5)	C8—C7—C6	130.9 (9)
C1—C2—C9	109.1 (5)	C8—C7—H7A	114.6
C3—C2—C9	114.0 (6)	C6—C7—H7A	114.6
C1—C2—H2A	110.6	C7—C8—H8A	120.0
C3—C2—H2A	110.6	C7—C8—H8B	120.0
C9—C2—H2A	110.6	H8A—C8—H8B	120.0
O3—C3—C2	111.7 (5)	C2—C9—H9A	109.5
O3—C3—C4	106.8 (5)	C2—C9—H9B	109.5
C2—C3—C4	102.5 (5)	H9A—C9—H9B	109.5
O3—C3—H3B	111.8	C2—C9—H9C	109.5
C2—C3—H3B	111.8	H9A—C9—H9C	109.5
C4—C3—H3B	111.8	H9B—C9—H9C	109.5
O1—C4—C5	109.1 (5)	C5—C10—H10A	109.5
O1—C4—C3	103.2 (4)	C5—C10—H10B	109.5
C5—C4—C3	117.6 (5)	H10A—C10—H10B	109.5
O1—C4—H4A	108.8	C5—C10—H10C	109.5
C5—C4—H4A	108.8	H10A—C10—H10C	109.5
C3—C4—H4A	108.8	H10B—C10—H10C	109.5
C4—C5—C6	112.3 (5)	C6—C11—H11A	109.5
C4—C5—C10	109.0 (5)	C6—C11—H11B	109.5
C6—C5—C10	112.8 (5)	H11A—C11—H11B	109.5
C4—C5—H5A	107.5	C6—C11—H11C	109.5
C6—C5—H5A	107.5	H11A—C11—H11C	109.5
C10—C5—H5A	107.5	H11B—C11—H11C	109.5
C7—C6—C11	115.1 (5)		
			
C4—O1—C1—O2	177.8 (6)	C2—C3—C4—O1	31.7 (6)
C4—O1—C1—C2	−3.8 (7)	O3—C3—C4—C5	34.3 (8)
O2—C1—C2—C3	−157.8 (7)	C2—C3—C4—C5	151.8 (5)
O1—C1—C2—C3	24.0 (7)	O1—C4—C5—C6	−54.5 (6)
O2—C1—C2—C9	81.5 (9)	C3—C4—C5—C6	−171.5 (5)
O1—C1—C2—C9	−96.7 (6)	O1—C4—C5—C10	179.7 (5)
C1—C2—C3—O3	80.8 (6)	C3—C4—C5—C10	62.7 (7)
C9—C2—C3—O3	−162.0 (5)	C4—C5—C6—C7	168.6 (6)
C1—C2—C3—C4	−33.2 (6)	C10—C5—C6—C7	−67.7 (7)
C9—C2—C3—C4	84.0 (6)	C4—C5—C6—C11	−62.3 (7)
C1—O1—C4—C5	−143.8 (5)	C10—C5—C6—C11	61.4 (7)
C1—O1—C4—C3	−18.0 (6)	C11—C6—C7—C8	5.6 (14)
O3—C3—C4—O1	−85.8 (5)	C5—C6—C7—C8	133.7 (11)

### Hydrogen-bond geometry (Å, °)

**Table:**

*D*—H···*A*	*D*—H	H···*A*	*D*···*A*	*D*—H···*A*
O3—H3···O2^i^	0.82	2.02	2.798 (6)	158

Symmetry codes: (i) −*x*+1, *y*−1/2, −*z*−1/2.
